# Supplementary material for: The impacts of conditions and person-organization fit on alliances performance: And the moderating role of intermediary
Source: PLoS One. 2022 Dec 7;17(12):e0275863. doi: 10.1371/journal.pone.0275863 (PMC9728903; doi:10.1371/journal.pone.0275863)
Supplement: Attachment — Submitted filename: Comments PLOSONE.docx [file pone.0275863.s002.docx]

The introduction section can be improved with standard english writing.

2. Second paragraph first line introduction citation is missing. Kindly cite the first sentence.

3. Fourth paragraph first sentence citation is missing. Kindly cite this sentence.

4. Clearly explain the gap in the current study in the introduction section second last paragraph and explain how this study contributes to the existing literature.

5. Literature review

section 2.1. First paragraph citations are missing. Kindly cite the relevant studies.

Section 2.2. Again discussion without citations. Kindly add relevant citations.

Section 2.3. Te authors discusses moderating effect of intermediary performance with only 3 citations in three paragraph. Kindly cite the justifications provided in the second and third paragraphs.

Chapter 3.

3.1 Research model: it is better to discuss about the relationships among construct in literature review chapter and insert the fugure at the end of 2nd chapter.

3.2. Questionnaire: What do you mean by tavel related industries? Kindly explain the related industries.

3.3. Data: What was the original sample size? How you determine the sample size? How many questionnaires were distributed. Kindly explain above points.

4. Results

 Since the study data collected was self-reported. Did you check common method bias? If yes, then how?

Table 1:

Alliance conditions standard factor laoding values are low when compared intermediary performance SFL but its CR value is 0.92. Contrary to this the CR value of intermediary performance is low with high standard factor loadings. How can you justify?

Kindly add table 4 summarise the hypotheses results. And also discuss individual hypothesis result and compare with past studies for better understanding of the readers. Extend and improve discussion section. In the current form discussion section is very weak.
